# Palynological data for the Late Glacial and Holocene (14.5–0 ka BP) from Füramoos, Southern Germany

**DOI:** 10.1016/j.dib.2021.107650

**Published:** 2021-11-27

**Authors:** Oliver A. Kern, Andreas Koutsodendris, Jörg Pross

**Affiliations:** Institute of Earth Sciences, Heidelberg University, Germany

**Keywords:** Holocene and late glacial, Vegetation, Paleoclimate, Paleoenvironment, Human impact, Anthropocene

## Abstract

We here present high-resolution palynological data from the Füramoos peat bog, located in the alpine foreland of Southern Germany. The data represent raw pollen counts of major arboreal pollen as well as agricultural indicator taxa for the Late Glacial and Holocene (14.5 ka BP to present) at Füramoos with an average temporal resolution of 100 years (50 years during critical intervals). The data are also provided as percentages, which are calculated based on the total sum of pollen grains, excluding pollen grains of sedges (Cyperaceae) and strictly aquatic taxa. The data yield insight into the vegetation dynamics in Central Europe in response to climatic and anthropogenic forcing, which are an integral part of the original research article (Kern et al., 2021). Considering its high temporal resolution and the robust age-depth model, the dataset is ideally suited to be included in regional syntheses of vegetation dynamics in Central Europe from the Late Glacial onwards. In addition to the data, we provide a detailed description of the Füramoos site and detail the palynological processing and analysing techniques used.

**Specifications Table**

**Table:** 

Subject	Earth and Planetary Sciences
Specific subject area	Palynology, paleoecology, paleoclimate
Type of data	Tables Figure
How the data were acquired	Three drillcores (FU1, FU3, and FU4) were acquired using a Wacker Neuson DH-65 drill hammer. A composite core was constructed using well-defined peaks in the palynological data, and samples were taken at an average spatial resolution of 2 cm. Dried samples were treated with heated NaOH and sieved through a 400 µm mesh. Residues were centrifuged and mounted on microscope slides using glycerol-gelatine. Microscope analysis was carried out with a Zeiss Axioscope.A1 using brightfield illumination and 400–1000x magnification.
Data format	raw
Description of data collection	A minimum of 300 pollen grains was counted per sample. The calculation of pollen percentages is based on the total sum of pollen grains excluding Cyperaceae (sedges), strictly aquatic taxa, and other non-pollen palynomorphs such as algae (e.g., *Pediastrum*) and spores (e.g., ferns and funghi).
Data source location	• Institute of Earth Sciences, Heidelberg University • Heidelberg, Germany • Coring locations for cores FU1, FU3, and FU4: 47°59′32.5 N, 9°53′13.9 E
Data accessibility	Repository name: PANGAEA DOI: https://doi.org/10.1594/PANGAEA.929748 Direct link: https://doi.org/10.1594/PANGAEA.929748
Related research article	Kern, O.A., Koutsodendris, A., Süfke, F., Gutjahr, M., Mächtle, B., Pross, J., 2021. Persistent, multi-sourced lead contamination in Central Europe since the Bronze Age recorded in the Füramoos peat bog, Germany. Anthropocene 36, 100310. https://doi.org/10.1016/j.ancene.2021.100310[1]

**Value of the Data**

• Our data provide a continuous, temporally highly resolved (∼100 yrs) palynological record for the Late Glacial and Holocene (14.5 ka BP to present) at Füramoos, Southern Germany. The data contribute to a better understanding of changes in past environments and ecosystems.
• The data capture the vegetation dynamics associated with the Late Glacial period (14.5–11.7 ka BP), and highlight the compositional turnover attributed to climate change during the Late Glacial, early and mid-Holocene (until c. 4.5 ka BP). For the late Holocene, our data document substantial anthropogenic impact on the terrestrial ecosystem at Füramoos.
• Our data are of particular interest for researchers involved in environmental dynamics, climate change, human impact, and the vegetation response to these factors.
• The data can be integrated into (supra-)regional syntheses of vegetation dynamics in order to investigate spatio-temporal patterns of environmental change in Europe throughout the Late Glacial and Holocene. Moreover, they can be helpful in reconstructing anthropogenic activities (e.g., agriculture and deforestation) in Southern Germany during the late Holocene.
• Arboreal pollen (AP) percentages are important indicators of the degree of forestation, which is influenced by a multitude of factors such as climatic or anthropogenic (e.g., deforestation) forcing. Agricultural indicator pollen (AIP) percentages allow to assess the human impact on the environment through agricultural activities.

## 1 Data Description

**Fig. 1:**
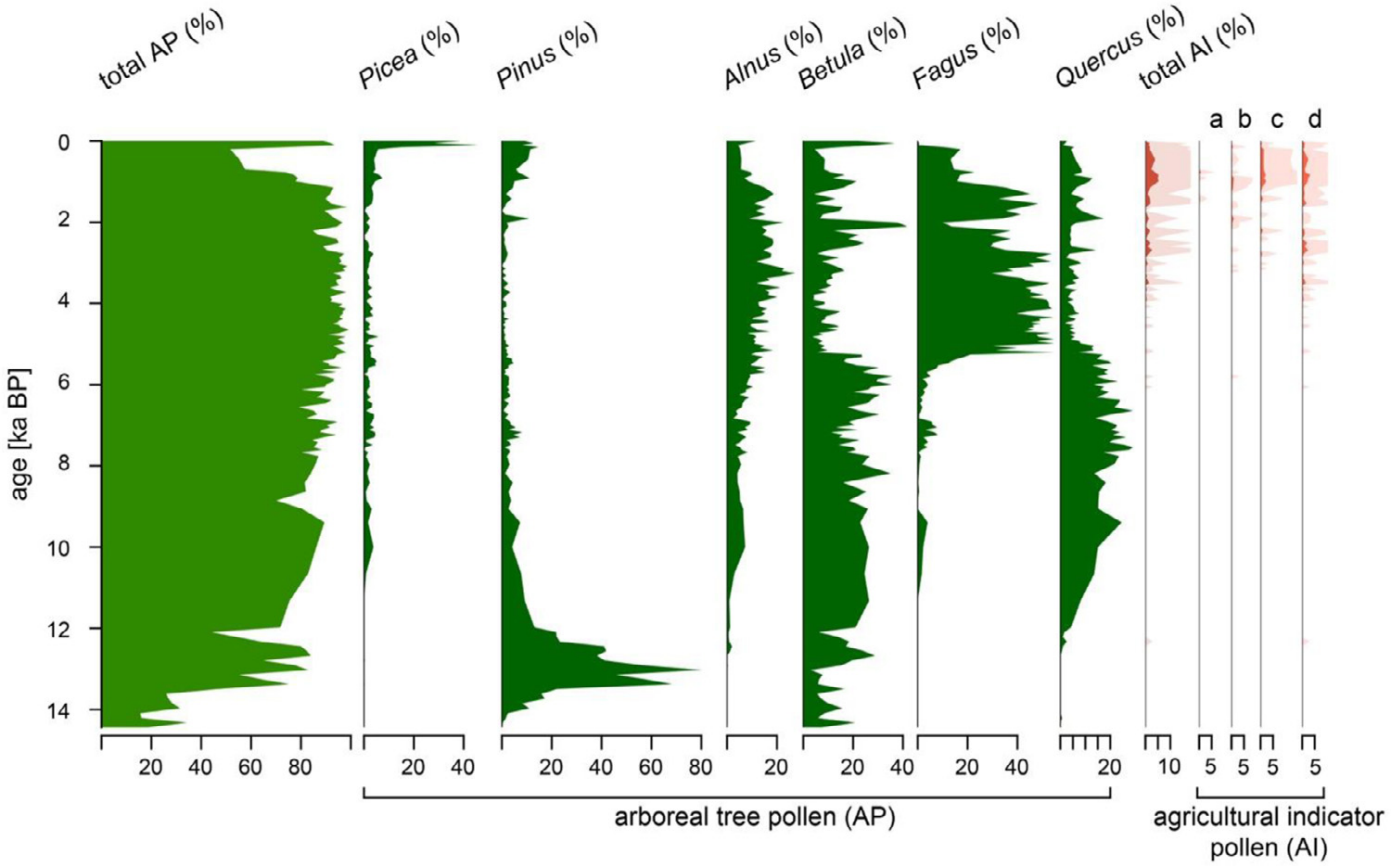
Arboreal pollen (AP) and selected other palynological data from the Füramoos composite core for the Late Glacial and Holocene interval (14.5 ka BP to present). Agricultural indicator pollen (AI) taxa include (a) *Castanea*, (b) *Juglans*, (c) Cerealia, and (d) Plantaginaceae. Shaded areas denote 10x exaggerated values.

We here present a continuous, high-resolution (mean: ∼100 years) palynological dataset comprising the Late Glacial (14.5–11.7 ka BP [before present; i.e., before 1950]) and Holocene (11.7 ka BP to present) at Füramoos, Southern Germany. The palynological dataset is provided in two separate excel sheets, one containing raw pollen counts and the other containing the pollen percentages calculated with respect to the total pollen counts for all taxa. Our data consist of major tree taxa (i.e., taxa with a mean representation of >5% throughout the interval) and agricultural indicator taxa (chestnut [*Castanea*], walnut [*Juglans*], cereal [Cerealia], and plantains [Plantaginaceae]) (Fig. 1). Moreover, we provide the sum of all tree pollen (AP) and the sum of all agricultural indicator pollen (AIP). Although plantains (i.e., *Plantago lanceolata*) are not cultivated, they are considered an important indicator of ley farming and fallow land, and are therefore included in the AIP as indicators of human activity [2].

## 2 Experimental Design, Materials and Methods

### 2.1 Study site

The Füramoos peat bog (662 m above sea level) formed in a small basin situated between two moraine ridges that formed during the Rissian glaciation (corresponding to MIS 6) in the northern alpine foreland of Southern Germany [3]. Its isolated location facilitated the near-continuous deposition of lacustrine sediments during the Last Glacial (MIS 5d to MIS 2) and peat deposits during interglacial periods (MIS 5e, 1). Its unique setting prevented the erosion of sediments by glacial- and glaciofluvial processes during the Last Glacial. Over the course of the Late Glacial (14.5–11.7 ka BP), the basin transitioned from a lake to a bog setting, which resulted in the deposition of an almost 3-m-long succession of ombrotrophic peat [4]. Ombrotrophic peat bogs are of particular interest for paleoenvironmental studies, because they receive their inorganic constituents solely from atmospheric sources and are therefore able to accurately record dust deposition and anthropogenic contamination [5]. As a result of peat cutting, basin drainage, and forestry in the early 18th to early 20th centuries [6], the formation of peat ceased at that time, and a boreal forest developed. Today, only relictual pockets of untouched peat that contain the entire sequence of Holocene deposits have remained at Füramoos [4].

### 2.2 Coring, chronology, and sampling

Three parallel drillcores (FU1, FU3, and FU4) were recovered using a Wacker Neuson DH-65 drill hammer at 47°59′32.5 N, 9°53′13.9 E during a drilling campaign in 2017 [7]. All cores were drilled within a horizontal distance of <1.5 m and were subsequently correlated using lithological marker horizons and well-defined changes in the palynological dataset. The latter effort resulted in a final composite core that has a total length of 2.76 m (0.12–2.88 m coring depth) and contains sections from all three cores. The age-depth model of the Füramoos composite record (Table 1) is based on 14 radiocarbon dates from bulk-peat samples [1] and two tie points (the beginning of peat harvesting [6] and the mid-point of the Younger Dryas). For a more detailed explanation of the construction of the composite core and the establishment of the chronology, we refer the reader to the original research article [1]. A total of 144 samples was taken from the composite core at a spatial resolution of 2 cm (yielding an average temporal resolution of ∼100 yrs based on the age-depth-model [1]) for palynological analysis; critical intervals were sampled at a resolution of 1 cm (∼50 yrs).

**Table 1:** Chronology of the Füramoos composite core, based on a combination of radiocarbon ages and tuning points [1].

composite depth [m]	^14^C BP	± BP	age [cal BP]	± cal BP	comment
0.20	–	–	200	–	tie point
0.22	776	24	698	23	^14^C dating
0.26	1068	24	988	59	^14^C dating
0.30	1679	23	1580	25	^14^C dating
0.40	2054	24	1997	51	^14^C dating
0.50	2446	24	2531	157	^14^C dating
0.60	2874	24	3007	49	^14^C dating
0.98	3925	20	4361	60	^14^C dating
1.30	4169	26	4736	89	^14^C dating
1.63	5036	23	5811	72	^14^C dating
1.78	5586	24	6361	40	^14^C dating
1.93	6112	24	7044	98	^14^C dating
2.23	6821	26	7651	26	^14^C dating
2.37	8215	38	9180	84	^14^C dating
2.46	–	–	12100	–	tie point
2.88	12400	40	14463	168	^14^C dating

### 2.3 Palynology

For each palynological sample, roughly 1 cm^3^ (0.2–0.5 g) of material was freeze-dried and spiked with one tablet of *Lycopodium clavatum* spores (Lund University, Batch No. 1031, 20848 ± 691 spores per tablet), which allows for an estimation of absolute palynomorph abundances (i.e., palynomorphs per cm^3^). Sample processing followed established protocols [8], although the nature of the sample material (ombrotrophic peat, >95% organic-matter content) allowed for a simplified routine: The removal of inorganic matter using hydrochloric acid (HCl), hydrofluoric acid (HF), and/or density separation using sodium polytungstate (SPT) was not required. Instead, the samples were left overnight in sodium hydroxide (NaOH, 10%) and then heated to ∼90–100 °C (just below the boiling point of the mixture) for 2 h. and were subsequently sieved through a 400 µm mesh to remove macroscopic fragments of organic matter. Residues were embedded in glycerol for storage. Sample aliquots were mounted on microscope slides using Kaiser's glycerol gelatine. Pollen analysis was performed with a Zeiss Axioscope.A1 microscope at 400–1000x magnification using differential interference contrast. A minimum of 300 pollen grains (range: 300–426) was counted for each sample. The identification of pollen grains followed [9]. The resulting data are provided as raw pollen counts and as percentages of the total sum of pollen counts. The calculation of pollen percentages is based on the sum of the individual taxa divided by the total sum of pollen counts, excluding pollen grains of Cyperaceae, aquatic taxa (e.g., *Ranunculus acris*), and other palynomorphs such as algae and spores.

## CRediT authorship contribution statement

**Oliver A. Kern:** Methodology, Investigation, Visualization, Writing – original draft. **Andreas Koutsodendris:** Conceptualization, Supervision, Writing – review & editing. **Jörg Pross:** Conceptualization, Supervision, Writing – review & editing.

## Declaration of Competing Interest

The authors declare that they have no known competing financial interests or personal relationships that could have appeared to influence the work reported in this paper.

## Appendix Supplementary materials

Image, application 1
